# Midkine is a potential novel marker for malignant mesothelioma with different prognostic and diagnostic values from mesothelin

**DOI:** 10.1186/s12885-017-3209-5

**Published:** 2017-03-23

**Authors:** Guntulu Ak, Yuji Tada, Hideaki Shimada, Selma Metintas, Masaaki Ito, Kenzo Hiroshima, Masatoshi Tagawa, Muzaffer Metintas

**Affiliations:** 1 Department of Chest Diseases, Medical Faculty, Lung and Pleural Cancers Research and Clinical Center, Eskisehir Osmangazi University, 26 000 Eskisehir, Turkey; 2 Department of Respirology, Graduate School of Medicine, Chiba University, Chiba, 1-8-1 Inohana, Chuo-ku, Chiba, 260-8670 Japan; 3 Department of Surgery, School of Medicine, Toho University, 6-11-1 Omorinishi, Ota-ku, Tokyo, 143-8541 Japan; 4 Department of Public Health, Medical Faculty, Lung and Pleural Cancers Research and Clinical Center, Eskisehir Osmangazi University, 26 000 Eskisehir, Turkey; 5 Department of Clinical Oncology, School of Medicine, Toho University, 6-11-1 Omorinishi, Ota-ku, Tokyo, 143-8541 Japan; 6 Department of Pathology, Tokyo Women’s Medical University, Yachiyo Medical Center, Yachiyo, 477-96 Owadasinden, Yachiyo, 276-8524 Japan; 7 Division of Pathology and Cell Therapy, Chiba Cancer Center Research Institute, 666-2 Nitona, Chuo-ku, Chiba, 260-8717 Japan; 8 Department of Molecular Biology and Oncology, Graduate School of Medicine, Chiba University, 1-8-1 Inohana, Chuo-ku, Chiba, 260-8670 Japan

**Keywords:** Mesothelioma, Mesothelin, Midkine, Prognosis, Biomarker

## Abstract

**Background:**

We evaluated possible diagnostic and prognostic values of serum midkine in malignant pleural mesothelioma in comparison with those of serum mesothelin, a well-established diagnostic biomarker.

**Methods:**

Serum mesothelin and midkine levels were determined with an enzyme-linked immunosorbent assay. We examined specimens from 95 Turkish cases with malignant pleural mesothelioma, 56 metastatic cancers to pleura, 27 other types of benign pleural diseases and 20 benign asbestos pleurisy. The cut-off values were 1.5 nmol/L for mesothelin and 421 pg/mL for midkine.

**Results:**

Sensitivity and specificity of mesothelin were 51.6 and 71.4%, 51.6 and 85.2%, and 51.6 and 85% for differentiating mesothelioma from metastatic cancers to pleura, other benign pleural diseases and benign asbestos pleurisy, respectively. Sensitivity and specificity of midkine were 61.1 and 41.1%, 61.1 and 48.1%, and 61.1 and 75% to distinguish mesothelioma from metastatic cancers to pleura, other benign pleural diseases and benign asbestos pleurisy, respectively. Combination of both biomarkers did not improve the differential diagnostic efficacy. Mesothelin levels were elevated in the epitheloid type and in the advanced cases, but were not related to the prognosis. In contrast, elevated baseline levels of midkine were independently associated with a poor prognosis of mesothelioma patients after adjusting for the stage, the histological subtypes and treatment schedules (HR = 1.84; 95% CI: 1.09-3.09) (*p* = 0.022).

**Conclusions:**

Serum mesothelin showed moderate sensitivity and high specificity to differentiate malignant pleural mesothelioma from metastatic malignancy to pleura and from benign pleural diseases. In contrast, midkine was a useful marker for predicting prognosis of mesothelioma patients.

## Background

Malignant pleural mesothelioma is an aggressive tumor with poor prognosis and remains a worldwide health problem. The diagnosis of mesothelioma is based on an immunohistochemical staining of pleural tissues obtained from biopsy [1]. Nevertheless, differentiation between mesothelioma and other pleural diseases is difficult in some cases even with the staining of biopsy specimens. In addition, there are not clinical and laboratory markers available which can predict prognosis of the patients. Development of such relevant markers is desirable not only for precise diagnosis but also for improving the treatment protocol. A less invasive method for collecting clinical specimens is also favorable for senior patients as are often found in mesothelioma.

Previous studies used serum and pleural effusions to search possible markers for diagnosis and prognosis of mesothelioma. Potential biomarkers that had been investigated included mesothelin [2–4], megakaryocyte-potentiating factor [5], osteopontin [6, 7], hyaluronan [8], syndecan [9], galectin-1 [10] and fibulin-3 [3, 11]. These biomarkers have moderate sensitivity but high specificity to differentiate mesothelioma from other pleural diseases. Two previous meta-analyses, for example, reported that both mesothelin and osteopontin showed 61 and 57% for the sensitivity and 87 and 81% for the specificity to differential diagnose mesothelioma from others, respectively [4, 7]. These are currently in use as positive diagnostic markers for mesothelioma in clinical fields. Nevertheless, negative results of these markers are not sufficient to exclude possibility of mesothelioma, and the positive results even need further validation with different diagnostic procedures. Subsequent studies also demonstrated that some of the above biomarkers could be related to the prognosis of mesothelioma [9, 10, 12, 13], but the value to predict the prognosis was limited.

There is not currently a reliable blood-based biomarker available as for diagnosis and prognosis of mesothelioma except serum mesothelin. Mesothelioma is one of the intractable cancers and is often resistant to chemotherapy. A good prognostic marker is thereby beneficial for the patients to select treatment options and furthermore, patient groupings based on such a marker will contribute to possible clinical studies that investigate efficacy of a therapeutic agent. Midkine is a heparin-binding growth factor that promotes survival, growth and migration of cells [14]. It is prominently expressed during embryogenesis, especially in the midgestation period, but is down-regulated to an insignificant level in healthy adults [15]. Overexpression of midkine has however been observed in various pathological conditions including cancer [16]. Midkine seems to play a crucial role in carcinogenesis and is markedly up-regulated in numerous types of malignancy [14]. Moreover, the midkine level can be correlated with a dim prognosis of several cancers [17, 18], but it has not yet been evaluated in mesothelioma. We investigated in the present study to compare mesothelin, a well-established biomarker for mesothelioma, with midkine, a possible novel marker, in the diagnosis and the prognosis of malignant pleural mesothelioma.

## Methods

### Patients and samples

A total of 198 patients who were diagnosed and treated in Eskisehir Osmangazi University Hospital, Turkey, were enrolled in this study. Three groups of patients included in the study were as follows: 95 patients with malignant pleural mesothelioma; 56 patients with metastatic cancers to pleura; 47 patients with benign pleural diseases including 20 with benign asbestos pleurisy and 27 with other benign pleural diseases. None of the patients had received chemotherapy, surgical treatments or radiotherapy prior to the diagnosis. Clinical data, including age, gender, histology, stage, treatment history, response to chemotherapy and survival characteristics, were collected for all the mesothelioma and other patients. All the procedures involving human participants were performed in accordance with the ethical standards of relevant committees in Eskisehir Osmangazi University, and with the 1964 Helsinki declaration and the amendments approved in Fortaleza, 2013. The local ethical committee of Eskisehir Osmangazi University approved the study, and all of the patients provided a written informed consent for analysis of biomarkers in their sera. The ethical committee of Chiba Cancer Center and Toho University also approved the study.

The diagnosis of mesothelioma and other cancers was confirmed with immunohistochemical staining. Benign asbestos pleurisy was diagnosed with a pathological analysis of pleural biopsy, a history of asbestos exposure and exclusion of the other causes of pleurisy, together with a 3-year observation period to eliminate possible malignancy. Other benign diseases included were tuberculosis, pulmonary embolism, congestive heart failure, hepatic hydrothorax, rheumatoid pleurisy, cholesterol pleural effusion, chronic renal failure, pneumoconiosis and parapneumonic pleurisy. These were diagnosed with a number of clinical characteristics and the histological analysis. The mesothelioma patients were staged according to the International Mesothelioma Interest Group staging system [19]. The response to the first-line chemotherapy, combination of cisplatin and pemetrexed, was evaluated using a modified response evaluation criteria in solid tumors (RECIST) [20].

Blood samples were sourced from the tissue bank of the Lung and Pleural Cancer Research and Clinical Center of Eskisehir Osmangazi University. The specimens were collected prospectively from the patients and stored at −80 °C.

### Assay for mesothelin and midkine

Serum mesothelin levels were determined with a chemiluminescent enzyme immunoassay using an anti-soluble mesothelin related peptide antibody (Fujirebio, Tokyo, Japan), and serum midkine concentrations were determined with an enzyme-linked immunosorbent assay using an anti-midkine antibody (Cellmid, Sydney, Australia), according to the manufacturers’ instructions. The assays were performed in an independent manner from the clinical data. Cut-off values for mesothelin and midkine were 1.5 nmol/L and 421 pg/mL, respectively. The cut-off value of serum midkine concentration, mean plus 2 times standard deviations (SD), was determined with data from Cellmid, which were based on 99 healthy blood donors. A concentration below 103.95 pg/mL was undetectable with the midkine assay.

### Statistical analysis

Data were analyzed using a statistical software (SPSS for windows, Version 15.0). Results were expressed as the mean value ± SD or median. The Kolmogorov-Simirnov test was used to assess distribution differences of the samples. Mann-Whitney U and Kruskal Wallis tests were used to compare 2 and more than 2 groups, respectively, since biomarker levels did not show normal distributions. Bonferroni correction was also used to determine difference of respective groups. Receiver operating characteristic (ROC) curve analysis was conducted to determine predictive values for mesothelin and midkine at differentiating mesothelioma from other pleural diseases. The area under the curve (AUC) and SD were also estimated. Predictive values were compared with the method of DeLong et al. [21]. The natural logarithm of biomarkers was used to compare predictive powers of the markers in combination, and then standardization of the markers was performed. The weight value of each marker was determined with the logistic regression analysis. Each marker was multiplied by the logistic regression coefficient, and the results were added to the combined marker values. The AUC, cut-off values and predictive values of new variables were then estimated. The median survival times and 95% confidence intervals (CI) were estimated for each mesothelioma group. The survival curves were generated with the Kaplan-Meier method. The median survival time between the groups was compared with the log-rank test. The Cox proportional hazards regression test was used to access effects of potential prognostic factors on survival. The survival was adjusted by several prognostic factors including the clinical stage, histological subtypes and treatment schedules.

## Results

### Patient profiles and serum biomarker levels

**Fig. 1:**
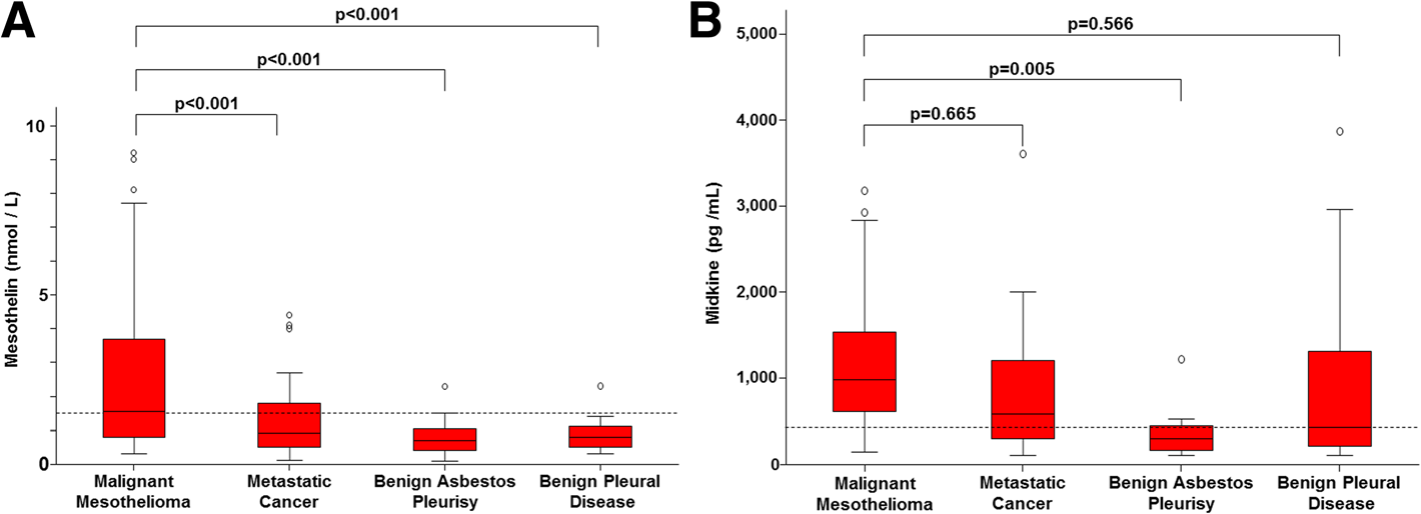
Serum concentrations of mesothelin (**a**) and midkine (**b**) in various diseases. Pleural diseases other than mesothelioma (shown in Table 2) are not included. Box plot with maximum and minimum values and the cut-off values (*dotted line*, mesothelin: 1.5 nmol/L, midkine; 421 pg/mL) are indicated. Statistical analysis was conducted with Kruskal Wallis tests

The profiles of the patients who were examined for serum concentrations of mesothelin and midkine were summarized in Table 1. Most of patients were from an area in Turkey where people were exposed to environmental asbestos, which made gender distribution of the patients almost equal. A majority of histological types in mesothelioma was epitheloid followed by mixed (biphasic) and sarcomatoid. We assayed 198 cases in total for the serum mesothelin and midkine concentrations, which included mesothelioma, metastatic cases to pleura, and benign diseases with and without asbestos pleurisy (Table 2). Serum mesothelin levels were significantly higher in the patients with mesothelioma than in those with metastatic cancers to pleura, benign asbestos pleurisy, benign pleural diseases or pleural diseases other than mesothelioma (Fig. 1a, Table 2). In contrast, serum midkine levels of mesothelioma patients were not different from those of patients with metastatic cancers to pleura, benign pleural diseases or pleural diseases other than mesothelioma, but were greater than those with benign asbestos pleurisy patients. (Fig. 1b, Table 2). Mesothelin therefore differentiated mesothelioma from non-mesothelioma diseases, but midkine had a limited value only to discriminate mesothelioma from benign asbestos pleurisy. Nevertheless, differential diagnosis between mesothelioma and benign asbestos pleurisy is quite important in clinical settings.

**Table 1:** Patient profiles

	Patient numbers (%)	Age average ± SD (min-max in year)	Gender Male/Female
Malignant mesothelioma	95 (48.0)	63.0 ± 11.0 (26-86)	49/46
Epitheloid	66 (69.5)		
Mixed (Biphasic)	15 (15.8)		
Sarcomatoid	8 (8.4)		
Undefined	6 (6.3)		
Metastatic cancers to pleura	56 (28.3)	61.6 ± 11.2 (34-85)	32/24
Lung cancer	38 (67.9)	62.2 ± 11.9 (34-85)	27/11
Other type of cancer^a^	18 (32.1)	60.4 ± 9.7 (39-77)	5/13
Non-malignant pleural diseases	47 (23.7)	55.8 ± 17.0 (19-81)	36/11
Benign asbestos pleurisy	20 (42.6)	61.7 ± 12.9 (40-81)	17/3
Benign pleural diseases^b^	27 (57.4)	51.4 ± 18.6 (19-77)	19/8
Total	198		

^a^including 8 cases of breast cancers, 3 lymphomas, 2 ovarian cancers, 1 stomach cancer, 1 renal cell carcinoma, 1 bladder cancer, 1 pancreatic cancer, 1 laryngeal cancer

^b^including 17 cases of tuberculous pleurisies, 2 pulmonary embolisms, 2 congestive heart failures, 1 hepatic hydrothorax, 1 rheumatoid pleurisy, 1 cholesterol pleural effusion, 1 chronic renal failure, 1 pneumoconiosis, 1 parapneumonic pleurisy

**Table 2:** Serum concentrations of mesothelin and midkine

	Mesothelin (nmol/L) Median (min-max)	Midkine (pg/mL) Median (min-max)
Malignant mesothelioma	1.50 (0.30-67.50)	656.04 (103.95-17,381.64)
Metastatic cancers to pleura	0.90 (0.10-9.70)	560.69 (103.95-35,742.24)
Benign asbestos pleurisy	0.85 (0.10-3.40)	538.06 (103.95-1217.00)
Benign pleural diseases	0.80 (0.30-67.50)	423.03 (106.25-37,459.76)
Pleural diseases other than malignant mesothelioma^a^	0.80 (0.10-9.70)	423.03 (103.95-37,459.76)

^a^Pleural diseases other than malignant mesothelioma include metastatic cancers, benign asbestos pleurisy and benign pleural diseases

### Diagnostic ability of serum biomarkers

**Fig. 2:**
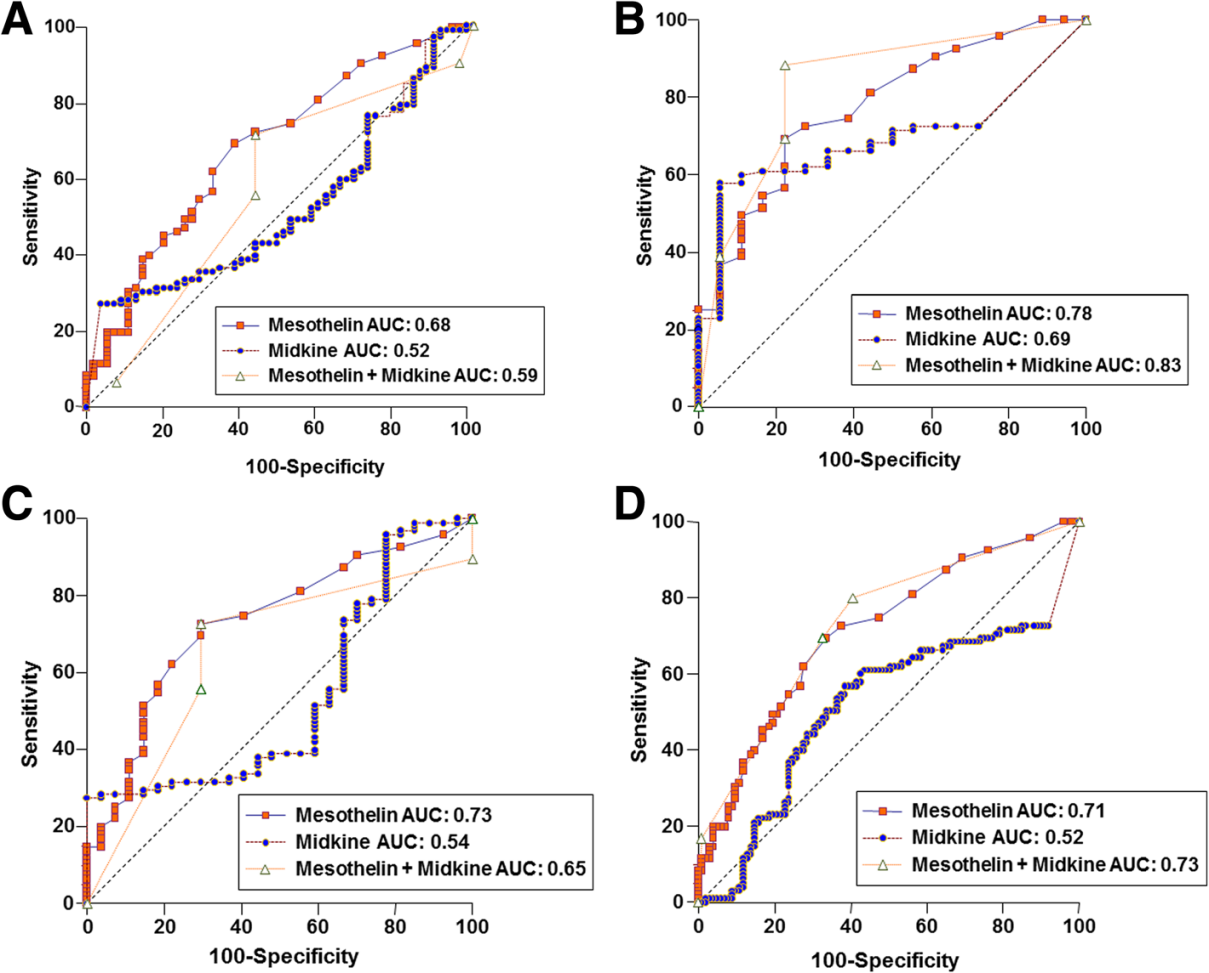
Receiver operating characteristic curve of serum biomarkers. The curve showed ability of mesothelin, midkine or the combination to differentiate between malignant mesothelioma and **a** metastatic cancers to pleura, **b** benign asbestos pleurisy, **c** benign pleural disease or **d** pleural diseases other than mesothelioma that include metastatic cancers, benign asbestos pleurisy and benign pleural diseases

We further investigated possible diagnostic values of mesothelin and midkine using ROC curve analyses to differentiate mesothelioma from non-mesothelioma patients (Fig. 2). The AUC of serum mesothelin to distinguish patients with mesothelioma from those with metastatic cancers to pleura was significantly higher than that of serum midkine (*p* = 0.0330) but was not different from that of combination of both mesothelin and midkine (*p* = 0.0738) (Fig. 2a). The AUC of serum midkine for differentiating mesothelioma from metastatic cancers to pleura was not different from that of the combination of the two markers (*p* = 0.3351). The AUC of serum mesothelin to distinguish patients with mesothelioma from those with benign asbestos pleurisy was not different from that of serum midkine (*p* = 0.1641) or that of the combination of the two biomarkers (*p* = 0.1055) (Fig. 2b). Nevertheless, the AUC of combined serum mesothelin and midkine to distinguish mesothelioma patients from benign asbestos pleurisy patients was significantly higher than that of midkine (*p* = 0.0329). The AUC of serum mesothelin to distinguish patients with mesothelioma from those with benign pleural diseases was significantly higher than that of midkine and the combination of the two biomarkers (*p* = 0.0576 and *p* = 0.0115, respectively) (Fig. 2c). The AUC of serum midkine to distinguish mesothelioma patients from benign patients with pleural diseases was not different from that of the combination of the two markers (*p* = 0.2476). The AUC of serum mesothelin to distinguish patients with mesothelioma from those with all the other diseases, including metastatic cancers, benign asbestos pleurisy and benign pleural diseases, was higher than that of midkine (*p* < 0.0001), whereas it was not different from that of the combination of the two biomarkers (*p* = 0.5598) (Fig. 2d). The AUC of the combination of the biomarkers was higher than that of midkine to distinguish patients with mesothelioma from all other patients in the study (*p* = 0.0002).

We also examined the sensitivity, specificity, positive and negative predictive values of mesothelin, midkine, and the combination of the biomarkers to differentiate patients of mesothelioma from other patients (Table 3). The sensitivity of midkine was higher than that of mesothelin, whereas the specificity of mesothelin was greater than that of midkine. The combination of both mesothelin and midkine showed a higher sensitivity than mesothelin or midkine alone, but specificity of the combination was similar to that of midkine except comparison of mesothelioma versus benign pleural diseases. The combinatory use of both markers thus had a limited diagnostic value compared with individual markers.

**Table 3:** Sensitivity, specificity, positive and negative predictive values of mesothelin and midkine in differentiating malignant mesothelioma from other diseases

	Malignant mesothelioma vs metastatic cancers	Malignant mesothelioma vs benign asbestos pleurisy	Malignant mesothelioma vs benign pleural diseases	Malignant mesothelioma vs Pleural diseases other than malignant mesothelioma
Mesothelin				
AUC (95% CI)	0.68 (0.60-0.75)	0.78 (0.69-0.85)	0.73 (0.64-0.81)	0.71 (0.64-0.77)
Sensitivity (%) (95% CI)	51.6 (43.6-59.6)	51.6 (42.5-60.7)	51.6 (42.7-60.5)	51.6 (44.6-58.6)
Specificity (%) (95% CI)	71.4 (64.2-78.6)	85.0 (78.5-91.5)	85.2 (78.9-91.5)	77.7 (71.9-83.5)
PPV (%) (95% CI)	75.4 (68.5-82.2)	94.2 (89.9-98.5)	92.5 (87.8-97.2)	68.1 (61.6-74.6)
NPV (%) (95% CI)	46.5 (38.5-54.5)	27.0 (18.9-35.1)	33.3 (24.9-41.7)	63.5 (56.8-70.2)
Midkine				
AUC (95% CI)	0.52 (0.44-0.60)	0.69 (0.60-0.78)	0.54 (0.44-0.63)	0.52 (0.45-0.59)
Sensitivity (%) (95% CI)	61.1 (53.3-68.9)	61.1 (52.2-70.0)	61.1 (52.5-69.8)	61.1 (54.3-67.9)
Specificity (%) (95% CI)	41.1 (33.3-48.9)	75.0 (67.1-82.9)	48.1 (39.3-57.0)	49.5 (42.5-56.5)
PPV (%) (95% CI)	63.7 (58.0-71.4)	92.1 (87.2-97.0)	80.6 (73.6-87.6)	52.7 (45.7-60.0)
NPV (%) (95% CI)	38.3 (30.5-46.1)	28.8 (20.5-37.1)	26.0 (18.2-33.8)	58.0 (51.1-64.9)
Mesothelin + Midkine				
AUC (95% CI)	0.59 (0.51-0.67)	0.83 (0.75-0.90)	0.65 (0.56-0.74)	0.73 (0.66-0.79)
Sensitivity (%) (95% CI)	88.4 (80.2-94.1)	88.42 (80.2-94.1)	100.0 (96.2-100.0)	80.0 (70.5-87.5)
Specificity (%) (95% CI)	32.7 (20.7-46.7)	77.78 (52.4-93.6)	0 (0.0-12.8)	58.8 (48.6-68.5)
PPV (%) (95% CI)	69.4 (60.4-77.5)	95.5 (88.7-98.8)	77.9 (69.5-84.9)	64.4 (55.1-73.0)
NPV (%) (95% CI)	62.1 (42.3-79.3)	56.0 (34.9-75.6)	0 (0-0)	75.9 (65.0-84.9)

*AUC* area under the curve, *PPV* positive predictive value, *NPV* negative predictive value

In addition, we evaluated possible utility of mesothelin and midkine to discriminate early-staged mesothelioma (stage I - II) from benign asbestos pleurisy since the differential diagnosis between the two diseases is clinically important. The AUCs of serum mesothelin and midkine to distinguish patients with the early-staged mesothelioma from patients with benign asbestos pleurisy were 0.655 (0.490-0.796), with a sensitivity of 42.9% and a specificity of 85%, and 0.557 (0.394-0.712), with a sensitivity of 33.3% and a specificity of 95.0%, respectively. Both of the markers could thus not discriminate early-staged mesothelioma from benign asbestos pleurisy (mesothelin; *p* = 0.0736, midkine *p* = 0.534).

### Clinical values of mesothelin and midkine in mesothelioma

We classified the serum marker levels of mesothelioma patients according to the stage, the histology and responses to chemotherapy (Table 4). Serum mesothelin levels tended to increase with the stage advancement (*p* = 0.052) and were higher in the epitheloid type than the other non-epitheloid types, which included mixed and sarcomatoid type, in histopathological classification (*p* = 0.033). Serum midkine levels also tended to increase according to the stage progress (*P* = 0.059), but the levels were not related to the histological types of mesothelioma (*P* = 0.501). Responses to chemotherapy judged with modified RECIST was evaluated with 45 out of 51 patients who received cisplatin plus pemetrexed-based chemotherapy, which included 16 patients (35.6%) with progressive disease, 17 patients (37.8%) with stable disease and 12 patients (26.7%) with objective response. Baseline serum mesothelin and midkine levels at the diagnosis were not related to the chemotherapy responses of mesothelioma patients.

**Table 4:** Serum biomarker levels of patients with malignant mesothelioma classified by stage, histology and chemotherapy responses

Classification	Mesothelin (nmol/L) Median (min-max)	Midkine (pg/mL) Median (min-max)
Stage (case number)^a^		
I (12)	0.95 (0.30-67.50)	220.54 (103.95-5508.08)
II (11)	1.20 (0.30-2.80)	513.61 (103.95-2143.51)
III (34)	1.25 (0.40-21.50)	644.89 (103.95-17,381.64)
IV (36)	2.90 (0.30-27.40)	925.17 (103.95-5244.74)
	*p* = 0.052	*p* = 0.059
Histology (case number)		
Epitheloid (66)	2.00 (0.30-67.50)	623.56 (103.95-17,381.64)
Non-epitheloid (23)	1.20 (0.30-9.0)	753.50(103.95-5244.74)
Undefined (6)^b^	1.25 (0.60-7.40)	768.13 (103.95-1722.46)
	*p* = 0.033	*p* = 0.501
Chemotherapy response (case number)		
Progressive disease (16)	1.75 (0.30-67.50)	731.47 (103.95-5508.08)
Stable disease (17)	1.70 (0.40-7.70)	417.94 (103.95-2832.48)
Objective response (12)	2.70 (0.70-21.50)	441.06 (103.95-17,381.64)
	*p* = 0.452	*p* = 0.669

^a^Two patients were not staged and excluded; ^b^Not analyzed because of insufficient case numbers

**Fig. 3:**
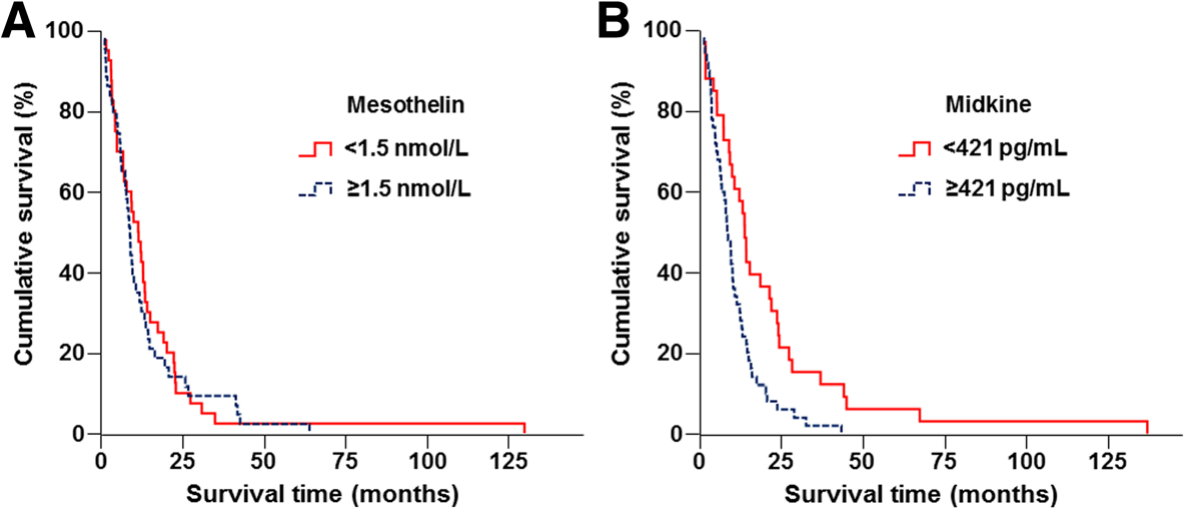
Kaplan-Meier survival curves of patients of mesothelioma. Survival of the patients classified by **a** mesothelin levels at 1,5 nmol/L and **b** serum midkine levels at 421 pg/mL

We then investigated possible prognostic values of serum mesothelin and midkine in patients with mesothelioma (Fig. 3). The survival was adjusted according to the clinical stage, histological subtypes and treatment schedules. Serum mesothelin levels were not related to median survival of patients with mesothelioma (*p* = 0.541) (Fig. 3a). In contrast, high serum midkine levels were independently associated with poor prognosis in mesothelioma (HR = 1.84; 95% CI: 1.09-3.09) (*p* = 0.022). The median survival time from diagnosis to death or the last day of follow-up with a 95% CI was 13.7 ± 1.2 months (95% CI: 11.33-16.13) for patients with serum midkine levels less than 421 pg/ml and was 8.4 ± 0.9 months (95% CI: 6.50-10.36) for those with serum midkine levels above 421 pg/ml (log-rank = 8.687; *p* = 0.003) (Fig. 3b). We did not notice any difference of the survival period between male patients, 10.1 ± 1.5 months (7.1-13.04), and female patients, 9.6 ± 1.7 months (6.28-12.99) (log-rank = 0.037; *p* = 0.847) in this study.

## Discussion

Mesothelin was regarded as a biomarker for differential diagnosis of mesothelioma [2–5, 12, 13, 22, 23]. In contrast, midkine has not yet been studied for a potential role in either the diagnosis or the prognosis of mesothelioma. In the current study, we compared the diagnostic ability of mesothelin and midkine to differentiate mesothelioma from other pleural disease, and we firstly demonstrated to our knowledge that serum midkine levels possessed a prognostic value in mesothelioma.

An initial study about mesothelin reported that an elevated serum concentration was indicative of mesothelioma with a sensitivity of 84% and a specificity of 100% in comparison with other pleural diseases and non-mesothelioma malignancy [2]. Subsequent studies however suggested that sensitivity and specificity of mesothelin in reference to various types of pleural diseases and lung cancer was not as great as reported in the initial study [3, 5]. A recent meta-analysis on serum mesothelin showed that the sensitivity and the specificity were 61 and 87%, respectively, when compared with various non-mesothelioma diseases [4]. In this study, we demonstrated that mesothelin levels in mesothelioma were greater than other pleural diseases when the serum concentrations was compared with respective diseases or the combination of all the diseases. The sensitivity and the specificity data in this study were consistent with previous studies [3, 5, 13, 22]. Other studies compared different kinds of biomarkers with mesothelin in terms of the diagnostic performance [3, 5, 12, 22, 23]. These markers, including megakaryocyte potentiating factor, fibulin-3, osteopontin, CA125 and hyaluronic acid, were not as accurate as mesothelin to differentiate mesothelioma from various non-mesothelioma diseases. We therefore evaluated midkine in the present study as a possible new marker for differential diagnosis. Sensitivity of midkine was comparable to or slightly greater than that of mesothelin, but specificity of midkine was lower than that of mesothelin. Midkine thus had less advantages than mesothelin as a diagnostic marker like other above-mentioned non-mesothelin markers except showing good specificity in differentiation from benign asbestos pleurisy. We however presume that a possible combinatory use of the non-mesothelin markers including midkine might attain a similar diagnostic level of mesothelin and such investigation needs to be conducted in future. Interestingly, Ostroff et al. screened more than 1000 serum proteins from mesothelioma patients and asbestos-exposed peoples with a proteomic assay, and listed midkine as one of the 13 markers in a panel that was useful for differential diagnosis of mesothelioma from others [24]. The study did not however show detailed data of midkine by itself in term of the diagnostic and prognostic values and the present study in fact firstly delineated the value of midkine as a biomarker in mesothelioma.

Detection of mesothelioma at an early stage is clinically important. A majority of mesothelioma patients however are diagnosed at an advanced stage and have poor prognosis with limited therapeutic efficacy. In clinical settings, most cases being suspected of mesothelioma can be diagnosed with a histological examination of pleural specimens. Differential diagnosis of mesothelioma from metastatic cancers to pleura and tuberculosis is not problematic, but benign asbestos pleurisy is the clinical entity that needs to be differentiated from an early-staged mesothelioma. In fact, mesothelioma at an early phase were sometimes diagnosed as benign asbestos pleurisy because the benign diseases are also associated with a history of asbestos exposure and the specimens showed non-specific immunohistochemical reactions. Consequently, a marker to differentiate between the two kinds of diseases is valuable for patients with either disease. A meta-analysis study dealing with respective patient data searched a possible merit of serum mesothelin in the diagnosis [25], which included more than 200 patients with stages I and II mesothelioma and 1600 symptomatic or high-risk controls. The results indicated that mesothelin showed a sensitivity of 32% at 95% specificity and furthermore about 70% of mesothelioma patients at the early stage were judged as negative for mesothelin. The poor sensitivity of mesothelin thus had a limited value for early diagnosis of mesothelioma. Our results also indicated that neither serum mesothelin or midkine discriminated mesothelioma at stage I or II from benign asbestos pleurisy. In contrast, a panel with 13 kinds of marker proteins which did not include mesothelin detected stage I and II mesothelioma with a sensitivity of 88% under 92% accuracy [24]. The data suggest that a combination of the markers, irrespective of whether mesothelin is included, can be useful for differential diagnosis at the early stage.

There are contradictory studies on mesothelin expression levels in different histological types and stages. A positive rate of mesothelin were greater in epitheloid type than in other types, and the mesothelin expressions increased according to advanced stage diseases [2], whereas other studies showed that mesothelin levels were not related to the histological type or stage of mesothelioma [3, 5, 12, 13]. The present study demonstrated that mesothelin levels preferentially elevated in epitheloid type compared with non-epitheloid type, and relatively increased with the advanced cases. In contrast, midkine levels were not different among the histological types, but they were associated with advanced staging with marginal statistical significance.

Several studies showed that mesothelin served as a marker for disease courses and for responses to the treatments [2, 26–28]. These studies indicated that increased serum levels of mesothelin were associated with disease progression and unresponsiveness to chemotherapy. Linch et al. however demonstrated that the mesothelin levels before chemotherapy did not correlate with the responses [29]. We examined the relationship between baseline levels of all the evaluable cases and their chemotherapy responses, and found that the levels of both mesothelin and midkine markers were not associated with the responses. The present results however needed careful evaluations since sensitivity to chemotherapy was different among the histological types and the clinical stages. We presume that a possible relationship between biomarker levels and chemotherapy responses should be examined in a prospective and a chronological manner. Previous studies also investigated any possible associations between baseline mesothelin levels and the prognosis of mesothelioma patients, and demonstrated that the mesothelin levels did not predict the prognosis [3, 28–30] although the tumor volume changes after chemotherapy were well correlated with mesothelin levels [31]. Other studies however reported contradictory results that an elevated mesothelin level was a poor prognostic factor in mesothelioma [12, 13]. In the current study, we demonstrated that mesothelin levels were not a predictive marker of the prognosis, whereas elevated midkine levels were linked to a poor prognosis. Other markers were also examined as for the prognostic values in mesothelioma patients, but osteopontin [12], hyaluronic acid [30] or fibulin-3 [3] were not a prognostic factor. In contrast, Hollevoet et al. showed that low baseline osteopontin was related with a favorable prognosis [28]. Treatment outcomes are influenced by many factors, which are not limited to histological types, clinical stages or performance status, and moreover these factors are often reciprocally associated with each other. These discrepant results about the prognostic values can thereby be derived from how these clinical parameters were analyzed.

Midkine expression in tumors was associated with cell proliferation because the transcriptional activity was dependent on cell growth and down-regulation of the expression decreased tumor cell growth [32, 33]. We showed that midkine was expressed in human mesothelioma cell lines (data not shown), but have not yet analyzed midkine levels in the specimens because precise estimation of tumor ratios in respective clinical samples is required. Relative low specificity of midkine as a marker is associated with lack of tumor type specificity of the midkine expression, and the elevated levels in metastatic cancers to pleura are attributable to the growth-linked property. Midkine expression is also linked to inflammatory reactions, which can contribute to elevated midkine levels in non-tumorous pleural diseases. Nevertheless, midkine levels were not elevated in benign asbestos pleurisy, in which inflammation was not probably involved. These data rather suggests that midkine is a negative maker to exclude benign asbestos pleurisy which must be differentiated from other pleural diseases including mesothelioma in clinical settings. In addition, these data collectively indicated that midkine was rather cell growth-related in contrast with mesothelin which is relatively tissue-specific.

We showed that a baseline midkine level had a prognostic value and the elevation was linked with poor prognosis. A few recent studies indicated that elevated midkine expression, assayed with immunohistochemistry or with the mRNA amounts, was a poor prognostic marker in lung carcinoma [17] and glioma [18]. Furthermore, Lv et al. suggested that cells with elevated midkine mRNA were resistant to cisplatin treatments [17]. In contrast, Wu et al. showed that midkine expression increased susceptibility of epithelial ovarian cancer cells to cisplatin/paclitaxel through down-regulated multidrug resistance-associated protein 3 and indicated that prognosis of the ovarian cancer patients with midkine-positive tumor cells was better than those with midkine-negative cells [34]. Extending tumor sizes due to the rapid proliferative ability is in general a poor prognostic factor but swift cell growth is also associated with increased sensitivity to anti-cancer agents targeting DNA replication and DNA damages. In clinical settings, many factors can affect drug sensitivity in vivo, which include genetic alterations of tumors such as those influencing anti-apoptotic and pro-apoptotic pathways. In addition, a serum concentration of proteins are regulated by a glomerular filtration rate and a body mass index of respective persons. These multiple factors in total reflect data of patient survival periods, and the prognostic values of a biomarker are subjected to an analyzing system how the patients are divided into subgroups.

## Conclusions

In conclusions, mesothelin is a useful biomarker with a moderate sensitivity and a relatively high specificity for the diagnosis of mesothelioma, but the level was not associated with the patient prognosis. In contrast, midkine can differentiate mesothelioma only from benign asbestos pleurisy but the base line level of midkine predicted prognosis of mesothelioma patients. Midkine is thus a novel biomarker for mesothelioma and has a different clinical value from mesothelin.

## Abbreviations

AUC: Area under the curve
CI: Confidence intervals
RECIST: Response evaluation criteria in solid tumors
ROC: Receiver operating characteristic
SD: Standard deviations
